# Two strains *Neocosmosporastercicola* (Sordariomycetes, Nectriaceae) with high nematicidal activity, isolated from the cysts of *Globodera* sp. (Heteroderidae) in China

**DOI:** 10.3897/BDJ.11.e100684

**Published:** 2023-04-27

**Authors:** Zaifu Yang, Hui Zhang, Zhaochun Jiang, Xinyue Zhang, Shan Wei, Yan Wu, Xiuhai Gan, Yong Wang, Xin Xie

**Affiliations:** 1 Institute of Vegetable Industry Technology Research, Guizhou University, Guiyang, China Institute of Vegetable Industry Technology Research, Guizhou University Guiyang China; 2 Department of Plant Pathology, College of Agriculture, Guizhou University, Guiyang, China Department of Plant Pathology, College of Agriculture, Guizhou University Guiyang China; 3 Guizhou Station of Plant Protection and Quarantine, Guiyang, China Guizhou Station of Plant Protection and Quarantine Guiyang China; 4 Center for Research and Development of Fine Chemicals, Guizhou University, Guiyang, China Center for Research and Development of Fine Chemicals, Guizhou University Guiyang China

**Keywords:** Plant-parasitic nematodes, *Neocosmosporastercicola*, nematophagous fungi, nematicidal activity

## Abstract

Plant-parasitic nematodes (PPNs) are significant pests that result in considerable economic losses in global crop production. Due to the high toxicity of chemical nematicides, there is a need to develop new strategies for nematode control. In this context, nematophagous fungi may offer a viable option for biological control. Two fungal strains (GUCC2212 and GUCC2232) were isolated from cysts of *Globodera* sp., identified as *Neocosmosporastercicola*. The fungal filtrates of the strains were evaluated for their nematicidal activity against three species of PPNs: *Aphelenchoidesbesseyi*, *Bursaphelenchusxylophilus* and *Ditylenchusdestructor*. The fermentation filtrates of two strains exhibited substantial toxicity towards the evaluated nematodes, with mortality rates reaching up to 100% within 72 h. Concurrently, *N.stercicola* also demonstrated predatory and parasitic behavior. The eggs of *Globodera* sp. were parasitized by the two strains. *N.stercicola* represents a newly recorded species in China and a novel nematophagous species. In conclusion, the two strains of *N.stercicola* show promise as biocontrol agents for PPNs management.

## Introduction

Plant-parasitic nematodes (PPNs) are important pests causing economically high yield losses in plants cultivated worldwide (Perry and Moens 2013). Agriculturally impactful nematode species include cyst nematodes (*Globodera* and *Heterodera* spp.), root-knot nematodes (*Meloidogyne* spp.), foliar nematodes (*Aphelenchoides* spp.), pine wilt nematode (*Bursaphelenchusxylophilus* Steiner and Buhrer, 1934), *Ditylenchusdestructor* Thorne, 1945, *Anguinatritici* Filipjev, 1936 (Abdel-Baset and Dawabah 2020, Xu et al. 2020, Kumar and Dara 2021). Among them, *Aphelenchoidesbesseyi* Christie, 1942, *B.xylophilus* and *D.destructor* are especially important in China. *A.besseyi* primarily infects rice, causing leaf damage characterized by white tips that progress to necrosis. Affected leaves curl and deform, inflorescences shrink and grain development is hindered, resulting in reduced yields (Kanzaki et al. 2012). In severe cases, yield losses can reach 60% (Bridge et al. 2005). *B.xylophilus* is responsible for causing many tree diseases globally (Filipiak et al. 2021) and is classified as a quarantine pathogen by most countries in the world (Evans et al. 1996). It is listed among the ten most harmful plant nematodes (Jones et al. 2013). It is mainly spread by the beetle *Monochamusalternatus* (Hope, 1842) feeding on healthy pine trees (Togashi and Shigesada 2006). When the pine wood nematode enters the stem of pine trees, it migrates through resin canals feeding on parenchyma cells, ultimately killing the host tree (Shinya et al. 2013). It is responsible for causing hundreds of millions of dollars in economic losses globally, including in Japan, the United States and Canada (Carnegie et al. 2018, Lee et al. 2021). *D.destructor* is also one of the most destructive plant pathogenic nematodes (Jin et al. 2018). It can parasitize more than 120 host plants in China and is one of the main pathogens of *Solanumtuberosum* and *Dioscoreaesculenta* (Huang et al. 2010), causing a 20% to 50% yield reduction and even 100% crop loss in endemic areas (Fan et al. 2015). In conclusion, the economic impact of these three plant nematodes in China and worldwide is substantial. As they affect various plant parts, including leaves, internal structures and tubers, they serve as representative PPNs for studying damage to different plant components. Consequently, effective control methods for these nematodes may also apply to other plant nematodes.

Nowadays, the global population continues to grow, there is an increasing demand for food, which necessitates the effective control of plant-parasitic nematodes(Poveda et al. 2020). However, in agricultural areas, nematicides are often used to prevent the damage of these nematodes. Due to the high toxicity of chemical nematicides, it is necessary to develop new control strategies against nematodes. In this respect, filamentous fungi can be an interesting biocontrol alternative (Zhang et al. 2014). They are able to reduce the damage caused by plant-parasitic nematodes directly by parasitism, antibiosis, paralysis and by the production of lytic enzymes. They also minimize harm by space and resource-competition, by providing higher nutrient and water uptake to the plant or by modifying the root morphology and/or rhizosphere interactions, that constitutes an advantage for the plant-growth (Iqbal et al. 2018). Therefore, the use of filamentous fungi as biological control agents is a promising durable biocontrol strategy in agriculture against plant-parasitic nematodes.

The fungal genus *Fusarium*, characterized by diverse morphology and ecology, is distributed worldwide in plants, plant products, air, water and soil. As a crucial group of plant-pathogenic fungi, *Fusarium* infects numerous crops, causing root rot, stem rot and other diseases that result in significant yield reductions globally (Summerell 2019, Jayawardena et al. 2020). However, some non-pathogenic *F.oxysporum* strain have been reported to enhance crop yields by suppressing nematode populations and reducing nematode-related damage (Kisaakye et al. 2022). *Fusarium* spp. have been associated with *Heteroderaglycines*, *Meloidogyneincognita*, *Globoderarostochiensis*, *G.pallida* and some species exhibiting nematicidal activity (Singh and Mathur 2010, Terra et al. 2018, Zhang et al. 2023).

The genus *Neocosmospora* (Hypocreales, Nectriaceae) has been segregated from the *Fusariumsolani* species complex (O'Donnell 2000). These fungi are widespread in soil, plant debris, living plant material, air, water and even animals, with some species being plant pathogens (Sandoval-Denis et al. 2019). *Neocosmospora* species have also been associated with *H.glycines* and displaying nematicidal activity against the second-stage juveniles and eggs of *H.glycines* (Chen et al. 2000, Hu et al. 2018). According to reports, *N.stercicola* is a less well-known species, has been found in soil and various plant hosts in Europe (Sandoval-Denis et al. 2019, Crous et al. 2021).

In this study, colonizing fungi were isolated from cysts of *Globodera* sp. in Weining County, Guizhou Province, China. Their nematicidal properties were evaluated by *in vitro* screening. Two strains (GUCC2212, GUCC2232) were found and its fermentation broth exhibited nematicidal activity against *A.besseyi*, *B.xylophilus* and *D.destructor*, mortality rates of 100%, respectively, after 72 h. The two strains were identified as *Neocosmosporastercicola* based on morphological observations and phylogenetic analysis of the genetic sequence of the internal transcribed spacer (ITS), large subunit (LSU) regions and translation elongation factor (*tef1*) of DNA. This is the first report of nematicidal activity against *A.besseyi*, *B.xylophilus* and *D.destructor* in the genus *Neocosmospora*. *N.stercicola* is the new record species in China.

## Material and methods

### Isolation of fungi from samples

Soil samples were obtained from a field of *Globodera* sp. infected potato fields located in Weining County, Guizhou, China. In each field, 10 plots of a 5 × 5 m grid were chosen around infected potato plants, and an approximate volume of 250 mL of soil from the rhizosphere zone was collected in each grid (0–20 cm depth). To make a single composite sample, the separate samples from each plot were collected and combined in a bucket (Southey 1974).

To ensure homogeneity, the composite samples were thoroughly mixed. Cysts of *Globodera* sp. were extracted from a subsample of 500 mL soil that had been air-dried at 37°C for two days (Been and Schomaker 2000, Reid and Pickup 2005, Nurjanah et al. 2016). Using the Baunacke method (Baunacke 1922, Hallman and Viaene 2013), cysts were obtained from a 100 g subsample of dried soil by decanting floating cysts in water and collecting them on a 250 mesh sieve.

Subsequently, the cysts were surface sterilized for three minutes using 0.2% H_2_O_2_, followed by three rinses with distilled water. Each sterilized cyst was individually placed on a 1% water agar (WA) plate. The plates were incubated at room temperature and monitored regularly. Mycelia emerging from the cultured cysts were transferred to new potato dextrose agar (PDA) plates multiple times. *N.stercicola* strains exhibiting high nematicidal activity were preserved in the Culture Collection of the Department of Plant Pathology, Agriculture College, Guizhou University, under strain numbers GUCC2232 and GUCC2212.

#### Collecting nematodes and culturing them

Samples of *A.besseyi* (identified by Zaifu Yang) were collected from rice fields in Dushan County, Guizhou Province, China and the Nematodes of *D.destructor* were provided by Nematode Laboratory of Fujian Agricultural and Forestry University. In this experiment, carrot callus were used to propagate the nematodes (Tulek et al. 2009). The nematodes were removed from the carrot-callus cultures and sterilized with streptomycin sulphate for 10 minutes, washed three times in double-distilled water and then cultured on carrot discs at 25°C for 30 d. *B.xylophilus* was provided by the Center for Research and Development of Fine Chemicals of Guizhou University. It was cultured on PDA plates containing *Botrytiscinerea* (Shi et al. 2013) and grown on the PDA plates at 28°C for 7 d.

*B.xylophilus* was transfered to *B.cinerea* plates and incubated at 28°C until the colony was consumed. By using the Baermann funnel method (Hallman and Viaene 2013), nematodes were separated from *B.cinerea* cultures. A nematode suspension was made at a concentration of 1000 nematodes/mL for use in the predatory and nematicidal activity assays of the fungal strains.

### Fermentation filtrate preparation

To prepare potato dextrose broth (PDB) medium, 200 g potatoes were boiled in 1 L of distilled water for 30 minutes. Dextrose was then added to the filtrate after the mixture was filtered through double gauze. Afterward, 100 mL aliquots of the prepared PDB medium were placed into 250 mL conical flasks and were autoclaved for 30 minutes at 121°C. With a sterilized cutting blade, the single pure culture was cut into small pieces approximately 5 mm in diameter. Five pieces were added in 100 mL of sterilized PDB medium, which was then shaken at 28°C for 7 d at 200 rpm on a rotary shaker. Finally, the medium was filtered and stored at 4°C.

### Morphological observations

The fungal strains GUCC2212 and GUCC2232 were inoculated on PDA and cultivated for 7 d at 28°C. Colony morphology was documented using a stereomicroscope (Keyence VHX-7000 digital microscope). A compound light microscope (Zeiss Scope 5) equipped with an AxioCam 208 color camera was used to photograph conidiophores and conidia. To determine the mean size, 30 conidiogenous cells, 50 macroconidia, and 50 chlamydospores from each strains were mounted and measured randomly.

### DNA extraction, PCR and sequencing

The fungal strains GUCC2212 and GUCC2232 were cultured on PDA for 7 d at 25°C. Using a sterile scalpel, the mycelia were carefully scraped from the plate's surface. Total genomic fungal DNA was extracted with a BIOMIGA Fungus Genomic DNA Extraction Kit (GD2416, BIOMIGA, San Diego, California, USA) following the manufacturer's instructions. The 5.8S nuclear ribosomal RNA gene was amplified and sequenced, together with the two flanking internal transcribed spacer (ITS), translation elongation factor (*tef1*) and large subunit (LSU) sections. The primer pairs and PCR amplification protocols ITS5/ITS4 (White et al. 1990a), EF1/EF2 (O'Donnell et al. 1998) and LR0R/LR5 (Perera et al. 2020) were used to PCR-amplify ITS, *tef1* and LSU, respectively.

PCR amplifications were conducted in a reaction mixture containing 12.5 µL 2x Bench Top Taq Master Mix (Biomiga, AT1201, China), 1 µL each of 10 µM primers and 1 µL DNA template, with the final volume adjusted to 25 µL using distilled deionized water. PCR products were visualized on a 1% agarose gel through electrophoresis. Bidirectional sequencing was performed by Sangon Biotech Company (Chengdu, China).

### Phylogenetic analysis

Using BioEdit v.7.2.5 (Hill 1999), forward and reverse primer sequences for each gene were assembled and consensus sequences were merged with relevant sequences obtained from the National Center for Biotechnology Information (NCBI). Table 1 presents the sequences of the two strains investigated in this study, along with 11 reference strains acquired from GenBank (https://www.ncbi.nlm.nih.gov/genbank). Sequences for each locus were aligned using MAFFT v. 7.187 (Katoh and Standley 2013) and alignments were manually adjusted as necessary.

Utilizing the maximum likelihood (ML) approach Bayesian inference (BI) via the CIPRES web platform, a phylogenetic tree was created using the ITS, *tef1* and LSU sequences as a concatenated dataset (Miller et al. 2010). Every 100 generations, trees were sampled and runs were automatically ended when the average standard deviation of split frequencies fell below 0.01. After removing the first 25% of samples, a 50% majority rule consensus tree was created. FigTree v1.4.3 (Rambaut and Drummond 2012) and Adobe Illustrator CC 2019 were used to visualize the generated tree.

### Infection of Globodera sp. eggs with fungi

To evaluate their pathogenicity against *Globodera* sp. eggs, pure fungal cultures were grown on PDA for 7–10 d. Conidial suspensions were prepared by flooding the PDA plates with double-distilled water (DDW) and the surface scraped. The suspension was diluted with DDW to achieve 1.0 x 10^6^ spores/mL using a haemacytometer.

For the egg parasitic ability test, eggs released from cysts by crushing them with a glass rod were suspended in DDW at a concentration of about 100 eggs per 100 µL suspension that was dropped into each well of a 24-multiwell plate containing each spore suspension with three replications. They were all incubated at 25°C and the number of eggs that were parasitized by fungi was examined every three days after incubation.

### Nematicidal activity of the fungal fermentation filtrate

A 100 μL suspension of nematodes containing approximately 100 nematodes was placed into wells of a 96-well culture plate containing different concentrations (100%, 20%, 10%) of the fermentation broth. The distilled water was added into the control wells. The plate was incubated for 72 h at 28°C. After 12, 24, 48 and 72 h, nematodes were examined under a microscope. The nematodes were cleansed and put into distilled water at each timepoint to assess their motility as a sign of nematicidal activity. When nematodes remained immotile after being probed with a fine hair needle, they were judged dead and percentage mortality was calculated. For each concentration and the control, four replicates were examined.

### Predatory activity of fungal isolates against nematodes in vitro

A 5 mm-diameter disc of mycelium was removed from the margins of the fungal isolates, placed to the center of a petri dish containing 1% water agar (WA) and cultured for two weeks in the dark at 25°C. Petri dishes were infected with a 1 mL nematode slurry containing 1000 nematodes after the incubation period. The nematode suspension was separated into 4-5 drops and evenly distributed across the fungal colonies periphery. Control plates were made without fungus. Each strain was tested in four replicates. A compound light microscope (Zeiss Scope 5) with an AxioCam 208 color camera was used to acquire microscopic images.

### Data analysis

The data were subjected to two-way analysis of variance (ANOVA), with concentration and post-treatment time (exposure period) serving as the main treatment effects and concentration x time as the interaction. Significant differences between means were determined at P<0.05 using Duncan’s multiple range. All statistical analyses were performed using MS Excel and SPSS statistics software (version 19.0). Figures were generated using Origin 2018.

## Results

### Fungi identification

**Figure 1.**
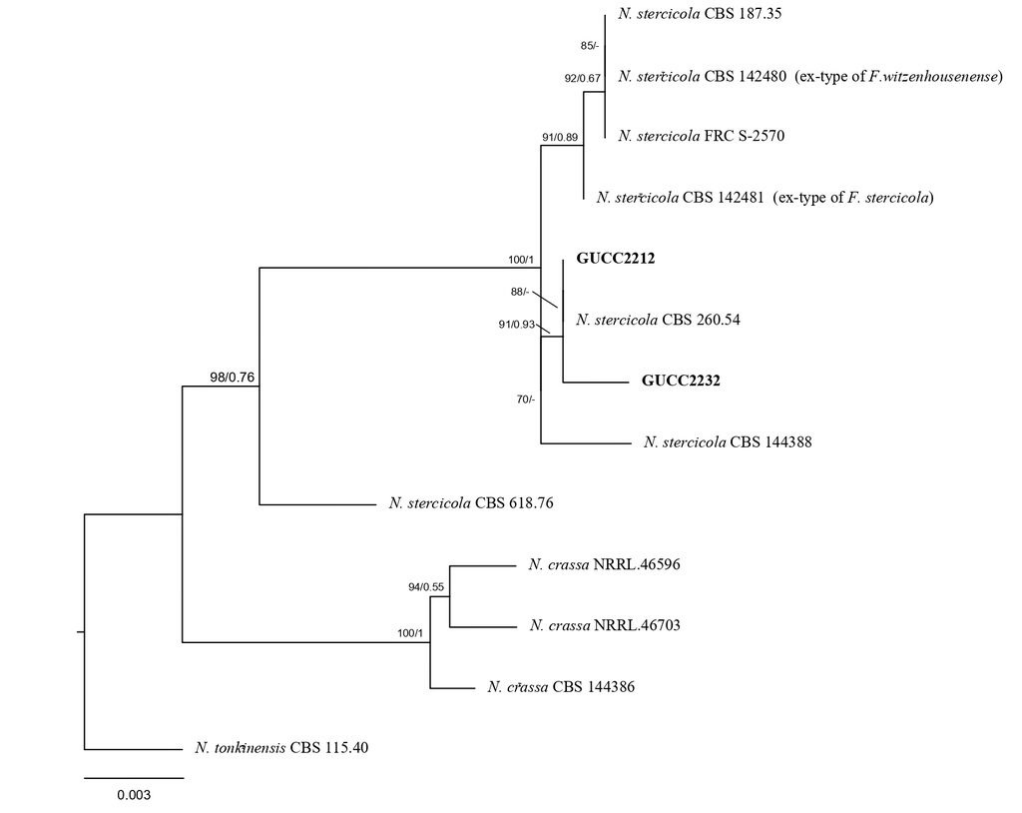
Phylogenetic tree generated from RAxML and Bayesian inference (BI) analysis based on combined ITS, *tef1* and LSU sequence data of *N.stercicola* (GUCC2232, GUCC2212). *N.tonkinensis* was designated as the outlier taxon. The scale bar indicates 0.003 nucleotide changes per site.

The sequences of the PCR products obtained from strain GUCC2232 and GUCC2212 were uploaded to GenBank and subjected to Basic Local Alignment Search tool (BLAST) analysis. In the phylogenetic tree (Fig. 1), GUCC2232 and GUCC2212 clustered with the type culture of *N.stercicola* (CBS 260.54). The ex-type cultures of species, *F.stercicola* and *F.witzenhausenense*, also cluster within this clade. Furthermore, the lack of clear distinctive morphological traits between these species, coupled with evidence of significant recombination within this clade according to the PHI test (Šišić et al. 2018), supports their synonymy as *N.stercicola*.

**Figure 2.**
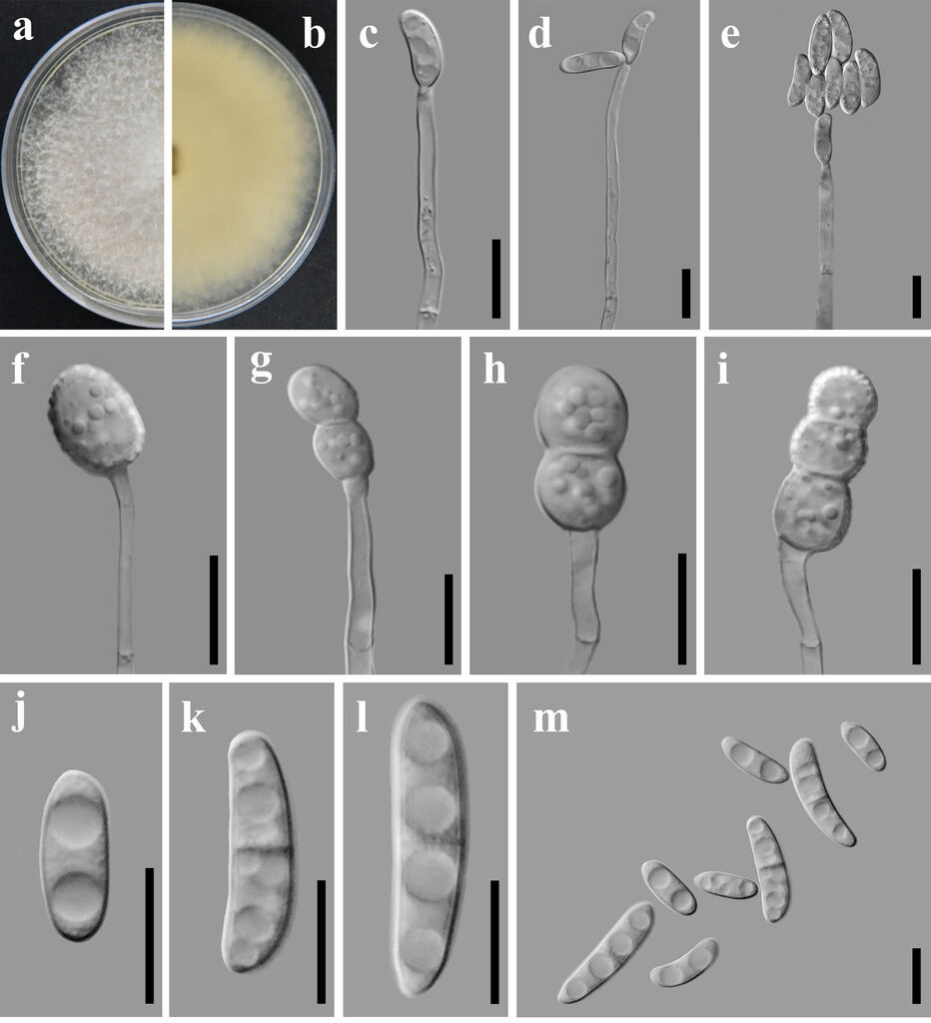
Colony and microscopic features of *N.stercicola* (GUCC2232). **a-b** Top and bottom images of GUCC2232 cultured on PDA **c** Monophialides and microconidia **d-e** short Monophialides with false head **f-i** Chlamydospores **j-m** multiseptate macroconidia. Scale bars: **c-g**, **i** = 50 μm; **h**, **j-m** = 10 μm.

The colonies of strain GUCC2232 on PDA after 7 d of incubation at 28°C exhibit a white to yellowish-white surface, with abundant cottony aerial mycelium throughout the colony. The reverse pigmentation is yellow at the center and fades to yellowish-white at the margin. The colony margin is undulate with an entire edge(Fig. 2a, b). The odour is strong moldy. Scant production of erect conidiophores, tapering uniformly from base to tip from the agar surface and aerial mycelium. Tip of the phialid with periclinical thickening, collared at most slightly flared (Fig. 2c, d, e). Conidia often held in clear, colourless drops of liquid, 0, 1, 2, 3 (- 4) septate: zero-septate ellipsoidal 11.40 × 4.12 μm; one-septate 19.27 × 5.25 μm; two septate 25.15 × 5.22 μm; three septate slightly curved, apical cell rounded and undeveloped foot cell 28.38 × 5.93 μm; 4 septate (n = 1) slightly curved with a slightly hooked apical cell and barely notched basal cell 27.29 × 6.13 μm (Fig. 2j-m). Chlamydospores formed abundantly after 7 d, unicellular, ovoid to ellipsoidal, single or in chains, terminal on hyphae or intercalary, consisting of enlarged, thick-walled vegetative cells: unicellular 6.29-6.84 μm; multicellular 10.85-11.57 μm in diam (Fig. 2f-i).

**Figure 3.**
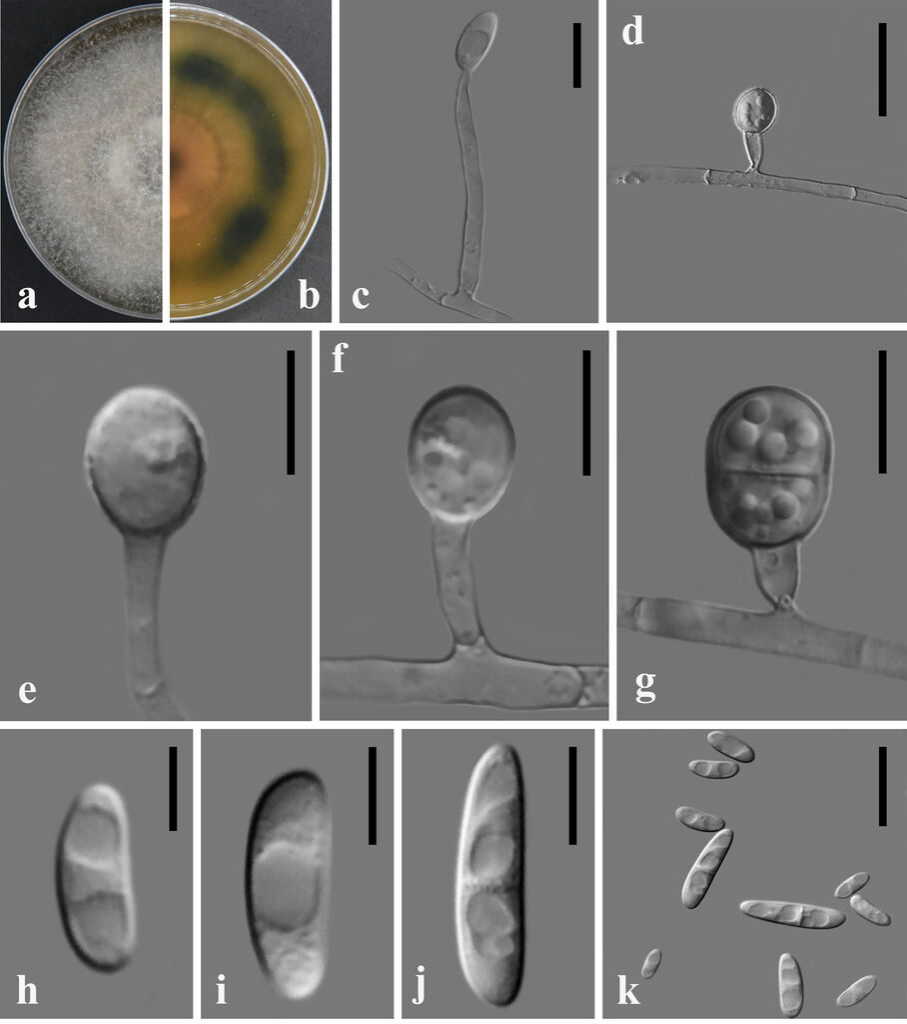
Colony and microscopic features of *N.stercicola* (GUCC2212). **a-b** Top and bottom images of GUCC2212 cultured on PDA **c** Monophialides and microconidia **d-g** Chlamydospores **h-k** short Monophialides multiseptate macroconidia. Scale bars: **c-g** = 50 μm; **i-k** = 10 μm.

The colonies of the GUCC2212 strain on PDA after 7 d of incubation at 28°C exhibited a brownish-grey center and yellowish-white margin, with radial V-shaped stripes composed of dense white flat mycelium stretching from the colony center to the edge. The aerial mycelium was scarce and the colony was fine with abundant production of cream slimy sporodochia at the margins. The reverse pigmentation was reddish-grey in the center, fading to yellowish white at the margin with irregularly distributed brownish-grey pigmented areas. The colony margin was entire to undulate (Fig. 3). The odour is sweet to moldy. The strain exhibited abundant production of erect, mononymous conidiophores from the agar surface and aerial mycelium. Tip of the Conidia often held in clear, colourless drops of liquid 0, 1 and 3 septate, with the zero-septate ellipsoidal or having a rounded apex and truncate base measuring 11.51 × 3.39 μm; one-septate ellipsoidal measuring 20.38 × 4.39 μm; and three-septate slightly curved or arcuate with a rounded apical cell and poorly developed basal cell measuring 28.05 × 5.95 μm (Fig. 3h-k).

Although the observed morphological characteristics of GUCC2232 and GUCC2212 were not entirely consistent with those described for *F.stercicola and F.witzenhausenense* by Šišić et al.(Šišić et al. 2018), we conclude that these strain belong to the *N.stercicola* species based on a combination of morphological observations and phylogenetic analysis.

### In vitro parasitism of fungal strains toward nematode eggs

**Figure 4.**
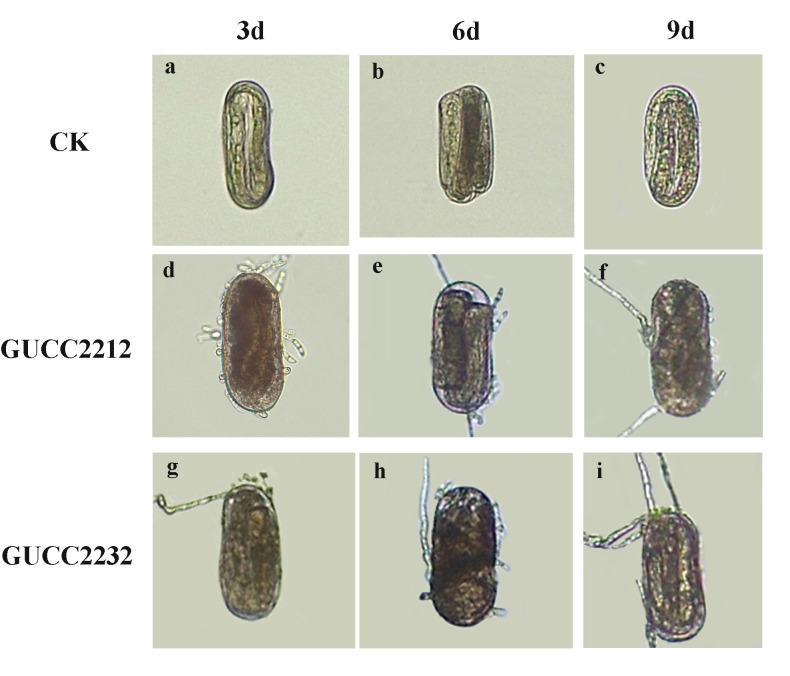
*Globodera* sp. eggs parasitized by the strains of *N.stercicola* (GUCC2212, GUCC2232). **a-c** Healthy egg **d** Hypha contact egg shell **e-i** Hyphae pass egg shell. Scale bars = 50 μm. Data represent the mean±SE (n = 4).

The fungal strains GUCC2212 and GUCC2232 were capable of infecting the eggs of *Globodera* sp. *in vitro*. Two strains infected and destroyed the *Globodera* sp. eggs (Fig. 4). At the same temperature with the same concentration, the parasitic rates differed at different times. The parasitic rate showed an overall upward trend with the extension of time and the parasitic rate of nematode eggs reached its highest 9 d after treatment. At this time, the conidia of *N.stercicola* were adsorbed or colonized on the shell surface of the nematode eggs and the hyphae surrounded the eggs and penetrated the eggshell (Fig. 4d-i), while no fungal growed from the control eggs (Fig. 4a-c). After 9 d of treatment with the same concentration of conidial suspension, the parasitical ratio of GUCC2212 was 21.19% and GUCC2232 was 22.41%, respectively. The relative parasitic rates of the two strains were very similar (Table 1).

### Nematicidal activity of the N.stercicola fungal fermentation filtrate

In order to obtain more microorganism resources to control plant-parasitic nematodes (PPNs), control efficiency of two fungal strains *N.stercicola* (GUCC2212) and *N.stercicola* (GUCC2232) on three PPNs were evaluated. The nematicidal activity of the *N.stercicola* fermentation filtrate was significantly different from that of PDB medium alone. PDB did not exhibit nematicidal or nematicidal activity because nematode mortality in PDB was statistically similar to that in sterilized water (P<0.05).

**Figure 5.**
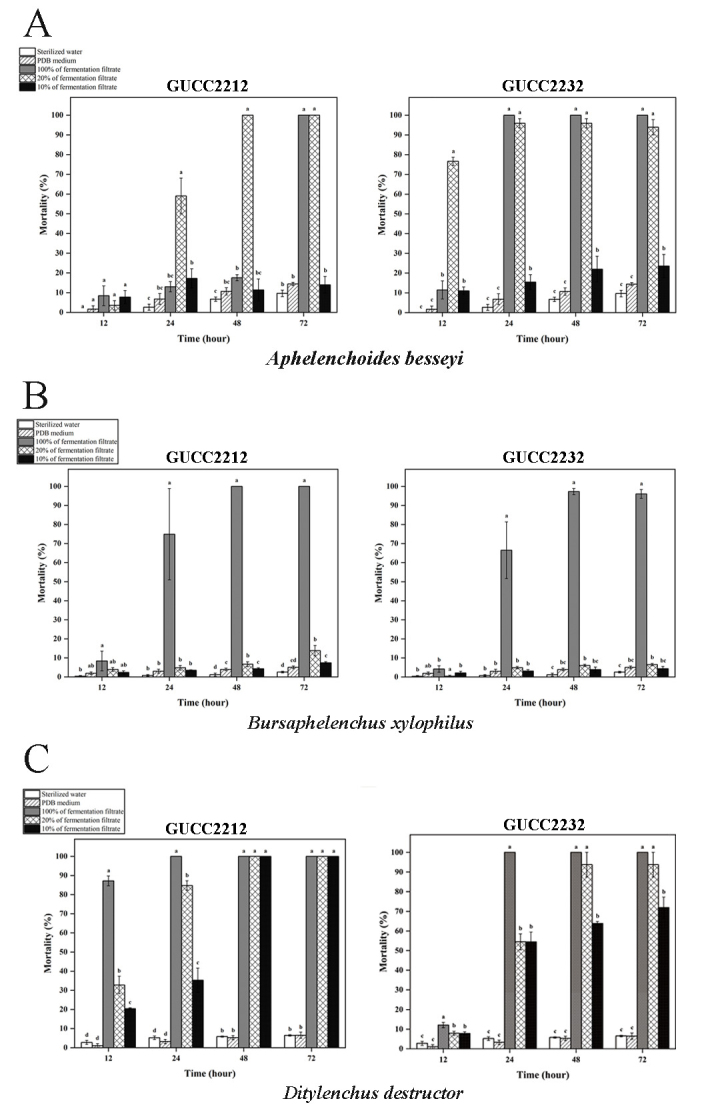
*In vitro* nematicidal activity of the fermentation filtrate of *Neocosmosporastercicola* (GUCC2212 and GUCC2232) against three species of plant pathogenic nematodes: **A** *A.besseyi* **B** *B.xylophilus* **C** *D.destructor*. *Note: Different letters indicate a significant difference between the different treatments within a given time point. Lowercase letters indicate significantly different means at P<0.05. Data represent the mean±SE (n = 4).

The mortality rate in *A.besseyi* was 12%, 100%, 100% and 100% at 12, 24, 48 and 72 h, respectively (Fig. 5A). The mortality rate in *B.xylophilus* increased rapidly, at 4%, 67%, 96% and 97% at 12, 24, 48 and 72 h, respectively (Fig. 5B).

Meanwhile, the nematicidal activity of the undiluted fermentation filtrate of *N.stercicola* (GUCC2212) was the highest nematicidal activity against *D.destructor*, with mortality rates of 47%, 90%, 96% and 100% at 12, 24, 48 and 72 h, respectively (Fig. 5C). Although the corrected mortality rate of nematodes after fermentation broth treatment should gradually decrease with increasing dilution factor, 10% preparation of the fermentation broth also exhibited a significant level of nematicidal activity at 72 h, relative to the controls against *A.besseyi*, *B.xylophilus* and *D.destructor* (P<0.05) (Fig. 5).

Although the two strains differed in their nematicidal activity against the three PPNs, collectively the data indicate that the fermentation broth of *N.stercicola* strains exhibited different levels of nematicidal activity against three different species of nematodes. Current investigations suggested that fermentation of *N.stercicola* could be used to control PPNs.

### In vitro predatory activity of N.stercicola against nematodes

**Figure 6.**
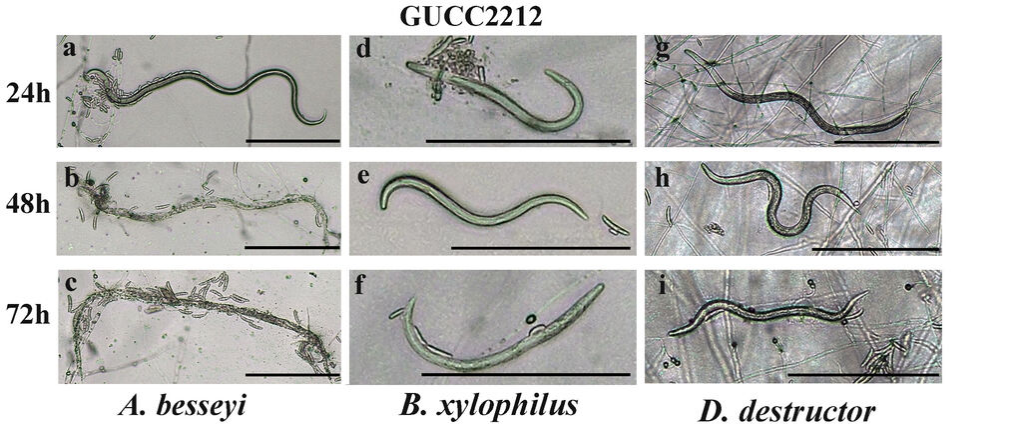
Predatory activity of *N.stercicola* (GUCC2212) against three plant pathogenic nematodes on water agar (WA). **a-c** Predatory activity against *A.besseyi* at 24, 48 and 72 h **d-f** Predatory activity against *B.xylophilus* at 24, 48 and 72 h **g-i** Predatory activity against *D.destructor* at 24, 48 and 72 h). Scale bars = 200 μm. Data represent the mean±SE (n = 4).

**Figure 7.**
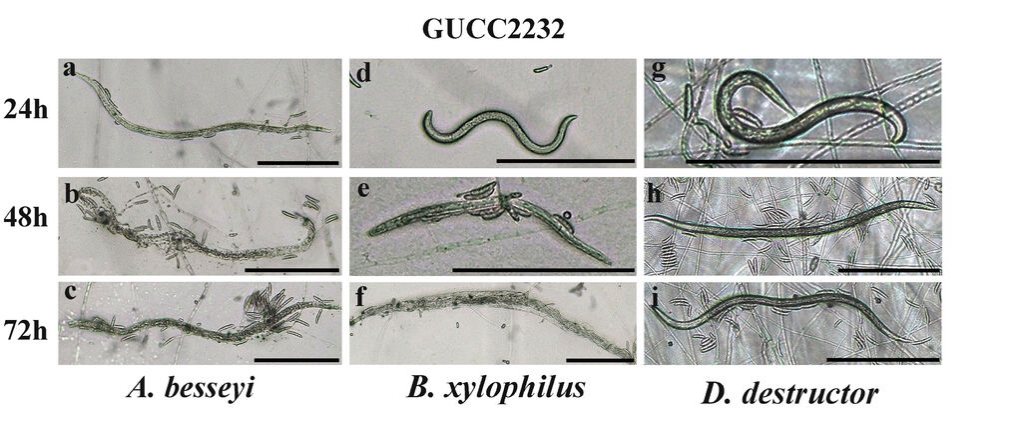
Predatory activity of *N.stercicola* (GUCC2232) against three plant pathogenic nematodes on water agar (WA). **a-c** Predatory activity against *A.besseyi* at 24, 48 and 72 h **d-f** Predatory activity against *B.xylophilus* at 24, 48 and 72 h **g-i** Predatory activity against *D.destructor* at 24, 48 and 72 h). Scale bars = 200 μm. Data represent the mean±SE (n = 4).

Nematodes moved freely on 1%WA plates of GUCC2212/GUCC2232 cultures during the initial 12 h of coincubation. However, their movement became increasingly limited between 12 and 24 h due to entanglement with hyphae. By 24-48 h of coincubation, nematodes were completely entrapped and unable to move. Two days later, the hyphae began to attach to the surface of entrapped nematodes, completely engulfing them. Following 72 h of coincubation, the number of fungal colonies increased, the body wall of the entrapped nematodes dissolved and their internal contents were consumed, leaving only traces of the entrapped nematodes behind (Fig. 6a-c, Fig. 7a-c).

Under *in vitro* conditions, the two strains of *N.stercicola* were cultured on 1% WA and separately co-incubated with three different species of nematodes. The number of total nematodes entrapped in five independent microscopic fields of view was recorded over 72 h. The results indicated that *N.stercicola* (GUCC2212, GUCC2232) exhibited predatory activity against nematodes at different points in time. In general, the percentage of trapped nematodes increased with the time of coincubation for *A.besseyi*. The predation activity of *N.stercicola* (GUCC2212,GUCC2232) was greatest against *A.besseyi*, with an average percentage of 35.19% and 37.96% respectively at 72 h (P<0.05) (Table 2).

## Discussion

Plant-parasitic nematodes (PPNs) are highly destructive endoparasites that infect a wide range of hosts and cause significant crop losses worldwide. Over-reliance on a single nematicidal agent has led to the emergence of new nematode races that are resistant to previous treatments, making it challenging to identify effective, safe and economical nematode management tools that do not harm non-target organisms. Biocontrol agents have emerged as a promising option due to their potential against target PPNs and safety for the environment. Among biocontrol agents, nematophagous fungi are particularly promising, as they utilize trapping devices from their vegetative mycelia and produce metabolites with nematicidal activity against infective juveniles.

To understand its importance in the integrated pest management programs and enrich the fungi options to be used as biocontrol agents, the present study was undertaken to characterize nematophagous fungal strains of *Neocosmospora*, which were isolated directly from cysts of *Globodera* sp. These two strains were used for species identification and evaluation nematicidal activity against PPNs.

Molecular characterization of the selected fungi was further supported by molecular characterization using three molecular markers. In the case of ITS, the sequence of *N.stercicola* (GUCC2212) showed a high similarity to the already reported strain of *N.stercicola* available in GenBank. Likewise, the sequence of *N.stercicola* (GUCC2232) also showed maximum identity to the already reported strain of *N.stercicola*. These findings confirm the identity and presence of *N.stercicola* in China. Our results showed that analysis of molecular variation and maximum composite likelihood analysis using ITS, LSU and *tef1* markers revealed a considerable degree of differentiation between geographical strains.

Previous studies have recorded the nematicidal activity of fermentation filtrates of some fungi, such as the study by Zhang et al. (Zhang et al. 2021) showed *Trichoderma* fertilizer≥2×10^8^ spore/g dustable powder had nematicidal activity against *D.destructor*, the corrected mortality rate reached 44.76% at 72 h. Similarly, *Beauveriabassiana* had a mortality rate of 64.85% against *A.besseyi* within 48 h (Zhao et al. 2013), while *Myrotheciumverrucaria* fermentation filtrates had a high nematicidal activity against *B.xylophilus*, with a mortality rate of 96.1% (Wu et al. 2020). In comparison, the nematicidal activity of *N.stercicola* (GUCC2212 and GUCC2232) fermentation filtrates against *A.besseyi*，*B.xylophilus* and *D.destructor* was significant, which the mortality rates reached up to 100% within 72 h. Although the level of nematicidal activity varied among the three nematodes, our data suggest that *N.stercicola* could be a new strain of fungus with potential for controlling PPNs. Compared with formerly reported commercial preparations of fungi, such as *Paecilomyceslilacinus* and *Verticilliumchlamydosporium* (Simon and Pandey 2010), which are primarily used as endoparasitic fungi to control the plant nematodes that damage plant roots, such as root-knot nematodes (Liu et al. 2021), our fungal metabolites have demonstrated a broad range of nematicidal activity based on *in vitro* studies.

According to the studies, Culture filtrate of *F.solani* and *N.vasinfecta* in malt extract broth show high nematicidal activity toward J2 of *H.glycines*, which paralyze percent reach 87% and 100% respectively in 72 h (Chen et al. 2000). Volatile organic compounds (VOCs) produced by *F.oxysporum* strain 21 with nematicidal activity against J2 of *M.incognita* and showed potential to be used in the field (Terra et al. 2018).

Two strains of *N.stercicola* (GUCC2212 and GUCC2222) have high nematicidal activity against three types of nematodes that damage different plant parts. Therefore, these strains are suitable for developing nematicides from the perspective of nematicidal modes and the targeting of nematodes. Further studies on the biology of the fungus and isolation of its nematically active substances are required.

Importantly, our study demonstrated the ability of *N.stercicola* to parasitize PPNs under *in vitro* conditions, making it a promising biocontrol agent. To the best of our knowledge, this is the first report of *N.stercicola* (GUCC2212, GUCC2232) exhibiting predatory activity against *A.besseyi* and *B.xylophilus* and parasitizing eggs of *Globodera* sp. The lower predation rate observed for *D.destructor* may be attributed to their fast movement, which could have prevented their immobiliation and paralysis. Overall, our study demonstrates the potential of *N.stercicola* as a biocontrol agent for nematode management.

## Conclusions

Despite numerous reports on the effectiveness of nematophagous fungi as biocontrol agents, the present study conducted in-depth research on the Chinese strains of *N.stercicola* for managing PPNs. In this study, we reported two strains of nematophagous fungus that were isolated from cysts of *Globodera* sp. Based on morphological observations and phylogenetic analysis of their ITS, *tef1*, and LSU DNA sequences, the two strains were identified as *N.stercicola*. This fungi is reported for the first time from China and show potential for PPNs management.

Commercially viable biopesticides can be developed based on the findings of the nematicidal pathway, such as a spore suspension or a nematicidal fermentation filtrate. For example, to prevent diseases caused by seed-borne organisms such as *A.besseyi*, seeds can be soaked before sowing or sprayed with the inoculant on the leaves and stems of rice. Spores or nematicidal fermentation filtrate can be injected for *B.xylophilus* parasitic nematodes in pine trees. For *D.destructor* and potato cyst nematodes parasitizing mainly bulbs, tubers, and root crops, root irrigation is one effective method in preventing diseases. However, further research is necessary to prove its efficacy in the field.

## Figures and Tables

**Table 1.** The parasitism rate of *N.stercicola* (GUCC2212, GUCC2232) against *Globodera* sp.

Treatment	Time		
	3 d	6 d	9 d
Control	0.00±0.00 a	0.00±0.00 a	0.00±0.00 a
GUCC2212	9.63±7.35a	13.82±2.49a	21.19±24.15a
GUCC2232	5.22±1.35c	13.25±1.79b	22.41±2.05a

**Table 2.** The predation rate of *N.stercicola* (GUCC2212, GUCC2232) against three species of plant pathogenic nematodes on water agar.

Target nematodes	Treatment	Time		
		24 h	48 h	72 h
*A.besseyi*	Control	1.11±0.56 a	2.41±0.49 b	3.98±0.65 b
	GUCC2212	2.55±1.62 a	10.37±3.88 a	35.19±2.73 a
	GUCC2232	1.72±0.87 a	22.87±3.40 a	37.96±1.34 a
*B.xylophilus*	Control	1.30±1.30 a	2.78±0.32 a	5.40±1.11 a
	GUCC2212	1.78±1.78 a	1.78±1.78 a	2.04±1.03 a
	GUCC2232	2.04±2.04 a	7.96±3.14 a	20.34±4.32 a
*D.destructor*	Control	0.00±0.00 a	0.00±0.00 a	0.00±0.00 a
	GUCC2212	0.00±0.00 a	0.00±0.00 a	0.00±0.00 a
	GUCC2232	6.67±6.67 a	13.33±6.67 a	15.00±7.64 a
